# Use of high-throughput RT-qPCR to assess modulations of gene expression profiles related to genomic stability and interactions by cadmium

**DOI:** 10.1007/s00204-015-1621-7

**Published:** 2015-11-02

**Authors:** Bettina Maria Fischer, Daniel Neumann, Ann Liza Piberger, Sarah Fremgaard Risnes, Beate Köberle, Andrea Hartwig

**Affiliations:** Department of Food Chemistry and Toxicology, Institute for Applied Biosciences, Karlsruhe Institute of Technology (KIT), Kaiserstrasse 12, 76131 Karlsruhe, Germany

**Keywords:** Genomic stability, Gene expression profiling, High-throughput RT-qPCR, Fluidigm dynamic array, Cadmium

## Abstract

Predictive test systems to assess the mode of action of chemical carcinogens are urgently required. Within the present study, we applied the Fluidigm dynamic array on the BioMark™ HD System for quantitative high-throughput RT-qPCR analysis of 95 genes and 96 samples in parallel, selecting genes crucial for maintaining genomic stability, including stress response as well as DNA repair, cell cycle control, apoptosis and mitotic signaling. The specificity of each individually designed sequence-specific primer pair and their respective target amplicons were evaluated via melting curve analysis as part of qPCR and size verification via agarose gel electrophoresis. For each gene, calibration curves displayed high efficiencies and correlation coefficients in the identified linear dynamic range as well as low intra-assay variations. Data were processed via Fluidigm real-time PCR analysis and GenEx software, and results were depicted as relative gene expression according to the ΔΔ*C*_q_ method. Subsequently, gene expression analyses were conducted in cadmium-treated adenocarcinoma A549 and epithelial bronchial BEAS-2B cells. They revealed distinct dose- and time-dependent and also cell-type-specific gene expression patterns, including the induction of genes coding for metallothioneins, the oxidative stress response, cell cycle control, mitotic signaling and apoptosis. Interestingly, while genes coding for the DNA damage response were induced, distinct DNA repair genes were down-regulated at the transcriptional level. Thus, this approach provided a comprehensive overview on the interaction by cadmium with distinct signaling pathways, also reflecting molecular modes of action in cadmium-induced carcinogenicity. Therefore, the test system appears to be a promising tool for toxicological risk assessment.

**Electronic supplementary material:**

The online version of this article (doi:10.1007/s00204-015-1621-7) contains supplementary material, which is available to authorized users.

## Introduction

During the last years, the need for large-scale tools in risk assessment of chemicals has been increasingly recognized (Rowlands et al. 2014; Thomas et al. 2013). Particularly, chemical carcinogens give rise to major concern. Since it is not suitable to perform long-term carcinogenicity studies as well as in-depth mechanistic studies for every single chemical of interest, predictive test systems are urgently needed. As carcinogens cannot be completely eliminated from workplaces, the environment or even food, the identification of modes of action, including the distinction between direct genotoxic and rather indirect acting carcinogens, is relevant for the assessment of dose–response relationships as a prerequisite for setting environmental and occupational exposure limits (Butterworth 1990; Silva Lima and Van der Laan 2000). In addition to genotoxicity and mutagenicity test systems, one promising approach consists in the evaluation of gene expression profiles provoked by the respective substances under investigation (Fielden and Zacharewski 2001; Waters et al. 2010), since changes in gene expression may serve as a sensitive and specific indicator for the toxic and genotoxic potential of substances. Thus, microarrays have been applied to reveal the impact of chemicals on genome-wide gene expression (Cunningham et al. 2000; Ellinger-Ziegelbauer et al. 2008; Gusenleitner et al. 2014; Nie et al. 2006; Nuwaysir et al. 1999; van Delft et al. 2004). Since results are only semiquantitative, they are usually confirmed by real-time RT-qPCR, which is very reliable but time-consuming and hence limited to few genes of interest. Nevertheless, during the last years attempts have been made to perform quantitative RT-PCR on a large scale. Thus, Fluidigm Corporation has developed microfluidic dynamic arrays for performing qPCR in a high-throughput format (Spurgeon et al. 2008), available in different designs. This approach has been applied for example for gene expression analyses on single-cell levels including tumor tissues (Citri et al. 2012; Diehn et al. 2009; Sanchez-Freire et al. 2012; White et al. 2011), for single nucleotide polymorphism genotyping (Wang et al. 2009) or for quantitative miRNA expression analyses (Jang et al. 2011; Petriv et al. 2010; White et al. 2011). One option of this array enables the parallel analysis of expression levels of 95 different genes and a “no-reaction control” for 96 different samples on the BioMark™ HD System, in which in each of the 9216 chambers of the array one distinct sample is combined with one primer pair specific for a target gene. Since the system is applicable for any set of genes, it can in principle be adapted to the respective question of interest.

Within the present study, we established a specific gene set using the 96 × 96 Dynamic Array™ to investigate the impact of different substances on expression levels of a selection of genes related to genomic stability. With respect to chemical carcinogens, in the past most emphasis has been given to the induction of DNA damage, which may lead to mutations and thus increase the risk of tumor development. In the meantime, it is evident that mammalian cells respond with manifold but highly coordinated reactions to genotoxic stress, referred to as the DNA damage response system. This includes the activation of DNA repair systems, but also cell cycle control, thereby increasing the time for DNA repair, as well as apoptosis eliminating heavily damaged cells (Harper and Elledge 2007; Zhou and Elledge 2000). The DNA damage response is strictly coordinated, for example, by the tumor suppressor protein p53 [reviewed in Hainaut and Hollstein (2000)]; it includes the activation of distinct signaling pathways which are up-regulated transiently upon the induction of cellular stress. Respective changes in gene expression are also described in tumor cells and tissues (Hanahan and Weinberg 2000, 2011), even though selection and/or adaptation within tumor tissues has to be taken into account.

We selected 95 genes of interest related to stress response as well as DNA repair, cell cycle control, apoptosis and mitotic signaling. To elucidate the power of this approach for the identification of interactions with signaling pathways related to genomic stability, we performed gene expression analyses with cadmium as one model substance with well characterized mode of action in A549 and BEAS-2B cells. Both cell lines are models for bronchial epithelia and have been selected since the lung is the major target organ of cadmium carcinogenicity upon inhalative exposure at workplaces and via smoking (IARC 2012). The experiments revealed distinct time- and concentration-dependent activations or repressions of genes related to uptake, oxidative stress response, anti-oxidative defense, mitotic signaling, apoptosis as well as DNA damage response and repair. Furthermore, they reflect molecular interactions involved in cadmium-induced carcinogenicity, pointing toward the great potential of this approach for the identification of modes of action of chemical carcinogens.

## Materials and methods

### Materials

Chemicals, including agarose, salts, glycerol, bromophenol blue, bovine serum albumin and acids, were obtained from Carl Roth GmbH (Karlsruhe, Germany). Cadmium chloride was purchased in high purity (>99.9 %) from Sigma-Aldrich Chemie GmbH (Steinheim, Germany). All PCR consumables including PCR tubes, strips and 96-well plates were obtained from Sarstedt (Nuembrecht, Germany). The primer pairs were synthesized by Eurofins (Ebersberg, Germany) or Fluidigm (San Francisco, USA). DMEM, trypsin, amphotericin B, trypsin inhibitor from glycine max (soybean) (SBTI) and penicillin–streptomycin solutions are products of Sigma-Aldrich. Fetal calf serum (FCS) and LHC-9 media are products of Invitrogen GmbH (Darmstadt, Germany). Human fibronectin was obtained from Biopur (Reinach, Switzerland) and collagen from Roche (Mannheim, Germany). Biochrom AG (Berlin, Germany) delivered cell culture dishes and flasks. DNA suspension buffer, PCR-certified water and TE buffer were obtained from Teknova (Hollister, USA). The 2× Assay Loading Reagent and 20× DNA Binding Dye Sample Loading Reagent were purchased from Fluidigm (San Francisco, USA). Bio-Rad (Munich, Germany) provided the 2× SsoFast™ EvaGreen^®^ Supermix with Low ROX and the 2× SYBR Green Supermix. The 2× TaqMan^®^ PreAmp Master Mix was obtained from Applied Biosystems (Darmstadt, Germany) and the exonuclease I from New England Biolabs (Frankfurt am Main, Germany). The IFC Controller HX and the BioMark™ HD System were purchased from Fluidigm (San Francisco, USA). The thermal cycler T100 and the qPCR system CFX96 were obtained from Bio-Rad Laboratories (Munich, Germany). Peqlab (Erlangen, Germany) delivered the PCR Workstation Pro.

### Cell culture and incubation

The adherent human adenocarcinoma cell line A549 was obtained from ATCC (ATCC CCL-185) and cultured as monolayer in DMEM containing 10 % FCS, 100 U/mL penicillin and 100 mg/mL streptomycin. Human lung bronchial epithelial BEAS-2B cells (ATCC CRL-9609), immortalized with SV40 large T-antigen, were kindly provided by Dr. Carsten Weiss (Karlsruhe Institute of Technology, Karlsruhe, Germany). They were grown as monolayers in coated cell culture dishes (10 µg/mL human fibronectin, 30 µg/mL collagen and 10 µg/mL bovine serum albumin in PBS) in LHC-9 medium containing 2.5 µg/mL amphotericin B. Cells were incubated at 37 °C in a humidified atmosphere of 5 % CO_2_ in air. Logarithmically growing cells were treated with CdCl_2_ as described for the respective experiments.

### RNA isolation and quantification

Total RNA from cell pellets was isolated with MN NucleoSpin^®^ RNA Plus KIT (Macherey–Nagel) according to the manufacturer’s instructions. RNA samples were stored at −80 °C for a maximum of 2 weeks. RNA content was determined by measuring absorption at 260 nm using a Nanodrop photometer (Tecan). Optimal purity of RNA was ensured by determination of the 260/280 adsorption ratio (values >2.00).

### RNA integrity

RNA integrity of A549 and BEAS-2B cells was confirmed by denaturing agarose gel electrophoresis using 1 µg total RNA with the ribosomal RNA species appearing as sharp bands. In intact RNA, the 28S rRNA band contains approximately twice the amount of the 18S rRNA band. Furthermore, RNA integrity was determined by a microfluidics-based electrophoresis system using a 2100 Bioanalyzer (Agilent Technologies). RNA integrity number (RIN) from automated analysis software allows classification of RNA in a numeric system with one for complete degradation and ten for optimal intactness. Both analyses displayed highly intact RNA, with RIN values of 9.8–10 (Supplementary material 5).

### Reverse transcription

One microgram of total RNA was reverse transcribed in duplicate per sample into first-strand complementary DNA (cDNA) using qScript™ cDNA Synthesis Kit (Quanta) according to the manufacturer’s instructions. cDNA samples were stored at −20 °C for a maximum of 4 weeks.

### High-throughput qPCR

High-throughput qPCR with Fluidigm dynamic arrays on BioMark™ HD System included several steps of sample preparation as recommended by the manufacturer’s instructions. All pipetting steps were carried out in a separate room under decontaminated and sterile conditions. DNase and DNA contamination was eliminated from laboratory surfaces by using DNA Away (Thermo Scientific). RNA- and DNA-free solutions were prepared in the PCR Workstation Pro (Peqlab).

#### Specific target amplification (STA)

To ensure adequate amounts of templates of the target genes for the high-throughput qPCR, a specific target gene amplification (STA) was performed. For STA, all sequence-specific primer pairs of the target genes were pooled and diluted with DNA suspension buffer to a final concentration of 500 nM (pooled primer mixture). Stock solutions of the pooled primer mixture were stored at −20 °C. A total of 5 µL STA mix was prepared containing 2.5 µL 2× TaqMan^®^ PreAmp Master Mix, 0.5 µL of the 500 nM pooled primer mixture, 0.75 µL PCR-certified water and 1.25 µL cDNA per reaction. A PCR-certified water control (NTC-STA) and a non-reverse-transcribed RNA (NoRT) control were also included. STA was performed in a thermal cycler (T100, Bio-Rad Laboratories) using the following temperature program: 10 min at 95 °C as an initial denaturation step followed by 12 cycles of 15 s at 95 °C for denaturation and 4 min at 60 °C for annealing and elongation and a final holding temperature of 4 °C. To prevent carry-over of unincorporated primers after the STA reaction, samples were treated with exonuclease I (*Escherichia coli*). Thus, 0.4 µL exonuclease I (Exo I) at 20 units/µL was diluted to 4 units/µL with 0.2 µL 10× exonuclease I reaction buffer and 1.4 µL PCR-certified water per reaction. To the STA samples, 2 µL of the exonuclease reaction mixture was added, and digestion with Exo I at 4 units/µL was performed in a thermal cycler with the following temperature program: 40 min at 37 °C for digestion of the unincorporated primers and dNTPs, 15 min at 80 °C to inactivate Exo I and a final holding temperature at 4 °C. STA and Exo I-treated samples were diluted fivefold with 18 µL TE buffer.

#### Preparation of samples and primers

Forward and reverse primers (100 µM) were diluted to 5 µM by adding 2.5 µL of each primer pair to 25 µL of 2× Assay Loading Reagent and 22.5 µL of DNA suspension buffer. The primer reaction mix was stored at −20 °C.

For the sample mix, 2.25 µL of STA and Exo I-treated samples were mixed with 2.5 µL of 2× SsoFast™ EvaGreen^®^ Supermix with Low ROX and 0.25 µL of 20× DNA Binding Dye Sample Loading Reagent.

#### Dynamic array IFC qPCR analysis

Preparation and loading of Fluidigm 96.96 Dynamic Array IFC (integrated fluidic circuit) was performed according to the manufacturer’s instructions. Briefly, preparation of the 96.96 Dynamic Array IFC included injection of 150 µL of a control line fluid into each accumulator of the chip with a syringe. After removal and discarding of the blue protective film from the bottom, the chip was placed into the IFC Controller HX and primed with the Prime (136×) script. After priming, the chip was loaded with samples, and the primer reaction mixes within 1 h to reduce the loss of the pressure within the chip. Thus, 5 µL of each primer reaction mix and each sample was pipetted into the respective inlets, avoiding the generation of air bubbles. Samples and primer reaction mixes were loaded into the chip by running the Load Mix (136×) script of the IFC Controller HX. After loading of the chip, potential dust particles were carefully removed from the surface of the chip using adhesive tape.

The chip was transferred into the BioMark™ HD System, and qPCR and melting curve analysis were performed by running the following temperature program: 2400 s at 70 °C and 30 s at 60 °C, followed by a hot start for 60 s at 95 °C, 30 PCR cycles of 5 s at 96 °C for denaturation and 20 s at 60 °C for annealing and elongation. The melting curve analysis consisted of 3 s at 60 °C followed by heating up to 95 °C with a ramp rate of 1 °C/3 s.

### Assessment of primer specificity

#### Conventional qPCR with 95 sequence-specific primer pairs

Primer specificity was evaluated by performing a conventional qPCR. One microliter of each pair of primers (10 µM) was mixed with 10 µL 2× SYBR Green Supermix, 1 µL commercial human standard cDNA (BioChain) and 8 µL PCR-certified water. For corresponding no-template controls (NTCs), human cDNA was replaced by 1 µL PCR-certified water. qPCR was performed in a real-time thermal cycler CFX 96 (Bio-Rad Laboratories) starting with 60 s at 95 °C, followed by 40 PCR cycles consisting of denaturation at 96 °C for 5 s, annealing and elongation at 60 °C for 20 s, a melting curve analysis of 3 s at 60 °C and finalized by heating up from 65 to 95 °C with a ramp rate of 1 °C/5 s.

#### Analysis of qPCR products by agarose gel electrophoresis

The gene amplification products were verified by size analysis via agarose gel electrophoresis. Five microliter of each qPCR product was mixed with 1 µL 6× loading buffer (25 mg bromophenol blue, 3 mL glycerol, 7 mL bidistilled water) and subjected to electrophoresis in a 3 % agarose 1× TAE gel containing Gel-Red (Biotium) for fluorescence detection (75 min, 100 V). Detection was carried out by the fluorescence imaging system LAS 3000 (Straubenhardt, Germany).

### Assessment of primer efficiency

Primer efficiency was determined via calibration curves with serial dilutions of commercial human cDNA as well as with cDNA derived from A549 cells via high-throughput RT-qPCR analysis with Fluidigm dynamic arrays using the BioMark™ HD System. Thus, 1–2 × 10^6^ logarithmically growing A549 cells were trypsinized, resuspended in ice-cold PBS containing 10 % FCS, collected by centrifugation, washed with ice-cold PBS and collected again by a second centrifugation step. Total RNA was isolated, quantified and reverse transcribed as described before. Subsequently, cellular A549 cDNA and human standard cDNA were diluted in quadruplicates 1:1, 1:5, 1:10, 1:50, 1:100, 1:150 and 1:200 in PCR-certified water before performing STA. Further analyses were performed as described before.

### Gene expression analyses with cadmium

0.5–1 × 10^6^ logarithmically growing A549 or BEAS-2B cells were treated with different concentrations CdCl_2_ for 8 or 24 h in DMEM containing 10 % FCS or in LHC-9 not containing FCS. After incubation, cells were trypsinized, resuspended in ice-cold PBS containing 10 % FCS (A549 cells) or containing 4 % SBTI (BEAS-2B cells), collected by centrifugation, washed with ice-cold PBS and collected again by a second centrifugation step. Total RNA was isolated, quantified and reverse transcribed as described before. High-throughput qPCR analysis with Fluidigm dynamic arrays was performed using the BioMark™ HD System.

### Data analysis and depiction

Initial data analysis for high-throughput RT-qPCR on BioMark™ HD System was accomplished with the software Fluidigm real-time PCR analysis. General scanning of the chip was performed with the passive reference dye ROX; respective files were examined and demonstrated equivalent ROX loading in all wells. Detection of the target amplicons and determination of the corresponding *C*_q_ values was performed with the fluorescent dye EvaGreen, which intercalates into double-stranded DNA. *C*_q_ values were determined with *C*_q_ threshold method “Auto detectors,” including a quality threshold of 0.65 and a linear baseline correction. *C*_q_ values were displayed as a results table, in which every reaction was listed with the corresponding numeric *C*_q_ value. Alternatively, they were displayed as an image view, in which the fluorescence signals for the respective dyes (ROX, Eva Green) according to the PCR cycle were shown in each well. Finally, they were visualized as a heat map, displaying genes in columns and the different samples in rows and visualizing *C*_q_ values in different colors according to a color key. Depiction of the *C*_q_ values in the heat map view allowed a global overview of the experiment as well as the identification of potential loading problems. Furthermore, the respective melting curves for every target amplicon of each sample were displayed by the software, providing the possibility to screen for unintended targets or the formation of primer–dimers. A quality check was always performed by including respective controls on each chip as stated above. Thus, the combination of the no-reaction control (NRC) without primers, the NTC and NTC-STA without cDNA and the NoRT control enabled the detection of potential contaminations in the reagents as well as reactions leading to the formation of unintended targets, primer–dimers and gDNA background. Further data analysis was performed with GenEx software after export of the full data set as heat map. Here, as a further quality control, a box plot of the “spread of genes” was performed for the identification of outliers. Next, a cutoff value of 22 was applied. Even though all negative controls displayed negligible signals, gDNA background was subtracted using *C*_q_ values obtained by the NoRT control. For normalization, five potential reference genes were available (*ACTB*, *B2M*, *GAPDH*, *GUSB* and *HPRT1*). The integrated programs geNorm and Normfinder were used to identify the optimal combination of reference genes for every experiment. geNorm compares the relative expression of pairs of genes in different samples and successively eliminates the gene with the highest expression variation ending up with an acceptable pair of reference genes applicable for normalization. *M*-values, which are related to the SD, are plotted for each potential reference gene and should not exceed values of 0.5 (Vandesompele et al. 2002). Normfinder applies a specific analysis of variance by calculation of a global average expression value for all genes, which is compared with the individual gene expression values resulting in the estimation of a SD, including also the different treatment groups. Consequently, any regulated or unstable gene can be identified and excluded as a reference gene (Andersen et al. 2004). By this procedure, suitable reference genes were identified for each experiment, which can differ between cell lines, as well as for different treatments. Finally, potential alterations of the transcript levels of the target genes under investigation were displayed as fold change compared with a control group by calculating relative quantities corresponding to the ΔΔ*C*_q_ method (Livak and Schmittgen 2001).

### Statistics

Differences between control and treated samples were analyzed by one-way analysis of variance (ANOVA) followed by Dunnett’s *T* post hoc test.

## Results

### Establishment of a gene set involved in genomic stability

Ninty-five genes were selected to investigate the modulation of cellular signaling pathways with high-throughput RT-qPCR on the BioMark™ HD System, based on careful consideration of criteria such as involvement in the maintenance of genomic stability and inducibility by exogenous and endogenous stressors. The selected genes are grouped in the following signaling pathways and cellular processes: (I) redox-regulated transcription factors, (II) proliferation and cell cycle control, (III) DNA damage response and repair, (IV) oxidative stress response, (V) apoptosis and (VI) xenobiotic metabolism (Table 1). The identities of the coding proteins for the respective genes are summarized in Supplementary material 1. The group of genes coding for selected redox-regulated transcription factors consisted of *TP53* (p53), *NFE2L2* (Nrf2), subgroups of NF-κB (*NFKB1*, *NFKB2*, *NFKBIA*) and *JUN* as factor of AP-1 as well as respective activators and inhibitors, which are supposed to be transcriptionally regulated by those transcription factors (reviewed in Angel et al. 1988; Harris and Levine 2005; Hoffmann et al. 2002; Kwak et al. 2002; Lustig et al. 2002). Proliferation-related genes were mitogens such as *MYC*, *EGFR* and *E2F1* as well as genes coding for cell-cycle-regulating proteins (cyclin D1 (*CCND1*) and cyclin-dependent kinase inhibitors (*CDKN1A*, *CDKN1B* and *CDKN2B*). The group of “DNA damage response and repair” covered genes coding for DNA damage signal transducers such as ATM and ATR and proteins involved in all major DNA repair pathways including nucleotide excision repair (NER), base excision repair (BER), mismatch repair (MMR) and DNA double-strand break repair (DSBR). To study “oxidative stress response,” genes coding for factors involved in ROS detoxification, e.g., the glutathione system, metallothioneins, other thiol-depending redox regulation systems like thioredoxin as well as anti-oxidative enzymes (SOD, CAT, HO-1) have been selected. The signaling pathway of apoptosis included genes coding for factors implicated in the extrinsic and intrinsic apoptotic pathway. Genes of phase I and II enzymes of biotransformation were part of the “xenobiotic metabolism” pathway. Additionally, five reference genes (*ACTB*, *B2M*, *GAPDH*, *GUSB* and *HPRT1*) for data normalization were included.

**Table 1:** Categorization of the 95 selected genes in distinct signaling pathways and cellular processes

Reference genes	Transcription factors	DNA damage response and repair	Apoptosis	Proliferation and cell cycle control	Oxidative stress response	Xenobiotic metabolism
*ACTB*	*AXIN2*	*APEX1*	*APAF1*	*CCND1*	*CAT*	*ABCB1*
*B2M*	*BTRC*	*ATM*	*BAX*	*CDKN1A*	*FTH1*	*ABCC1*
*GAPDH*	*JUN*	*ATR*	*BBC3*	*CDKN1B*	*G6PD*	*ADH1B*
*GUSB*	*KEAP1*	*BRAC1/BRCA2*	*BCL2*	*CDKN2B*	*GCLC*	*ALDH1A1*
*HPRT1*	*MAP3K5*	*DDB1/DDB2*	*BCL2L1*	*E2F1*	*GPX1*	*CYP1A1*
	*MDM2*	*DDIT3*	*PMAIP1*	*EGFR*	*GPX2*	*EPHX1*
	*NFE2L2*	*ERCC1/ERCC2*	*TNFRSF10B*	*IL8*	*GSR*	*GSTP1*
	*NFKB1*	*ERCC4/ERCC5*	*XIAP*	*MYC*	*HMOX1*	*NAT1*
	*NFKB2*	*GADD45A*		*PLK3*	*HSPA1A*	*NQO1*
	*NFKBIA*	*LIG1/LIG3*		*PPM1D*	*MT1X*	*SULT1A1*
	*TP53*	*MGMT*		*SIRT2*	*MT2A*	*UGT1A*
	*SLC30A1*	*MLH1*			*PRDX1*	
	*VEGFA*	*MSH2*			*SEPP1*	
		*OGG1*			*SOD1*	
		*PARP1*			*SOD2*	
		*PCNA*			*TFRC*	
		*POLB*			*TXN1*	
		*POLD1*			*TXNRD1*	
		*RAD50/RAD51*				
		*RRM2B*				
		*XPA/XPC*				
		*XRCC5*				

### Design of the sequence-specific primer pairs for the selected genes

Primer pairs for the respective genes were in part designed and derived from Fluidigm and in part designed by our group applying the Beacon Designer 8 software. The following criteria were chosen for primer design: exon–exon-junction-spanning primers or intron-spanning primer pairs with a minimum intron length of 700 bp, avoidance of cross-homology to other genes and avoidance of the formation of internal secondary structures of the target gene and the primers at the corresponding annealing temperature of 60 °C. Since RNA sequences of few genes comprised only short introns or even no introns at all, the designed primer pairs for six genes (*MT1X*, *SLC30A1*, *CDKN2B*, *GPX1*, *HSPA1A* and *JUN*) failed the criteria of spanning an intron at all and primer pairs for 11 genes (*BCL2L1*, *MT2A*, *NFKBIA*, *ACTB*, *CDKN1B*, *CYP1A1*, *ERCC2*, *G6PD*, *GSTP1*, *NQO1* and *POLD1*) covered an intron less than 700 bp. Nevertheless, specificity of the mentioned primer pairs was ensured by validation. Further criteria for the primer design included melting temperature (60 °C), length (18–24 nucleotides), GC content (40–60 %), avoidance of self-complementarity especially at the 3′ ends of the primer pairs and a defined amplicon length of 60–250 bp. Gene sequences were imported from NCBI (NCBI 2014). The selected transcript variant for primer design was mostly the dominant one, and for nearly all genes, the designed primer pairs were capable of capturing most or all relevant gene transcript variants. The gene symbol, gene ID, the reference sequence accession number (RefSeq), primer sequences, location of the primers and the confirmed targeted splice variants are listed in Supplementary material 2. Additionally, an in silico specificity screen was performed applying the Primer-BLAST software from NCBI for verifying specificity of the designed primer pairs against the whole human RefSeq mRNA database. Here, the stringency for the primer specificity was two total mismatches to unexpected targets, including at least two mismatches within the last 5 bp at the 3′ ends of the primer, whereas unintended targets with five or more mismatches to the primers were ignored. Twelve primer pairs failed this mathematical specificity check revealing potential unintended amplicon products. However, for five genes, the unintended targets could be identified as amplicons of distinct isoforms of the respective target genes themselves instead of by-products (*MT1X*, *SULT1A1*, *TNFRSF10B*, *UGT1A* and *VEGFA*). Since the mRNA sequence of those isoforms displays only slight differences in the nucleotide sequence, they can be captured by the primer pairs as well. For the remaining genes (*AXIN2*, *FTH1*, *BRCA2*, *CCND1*, *ERCC2*, *G6PD* and *GADD45A*), the potential resulting unintended amplicons differed very much in length from the intended target amplicon of the gene of interest (>1000 bp as opposed to up to 250 bp for the intended amplicons) and were thus easily excluded by size (see below).

### Determination of PCR assay specificity and performance

**Fig. 1:**
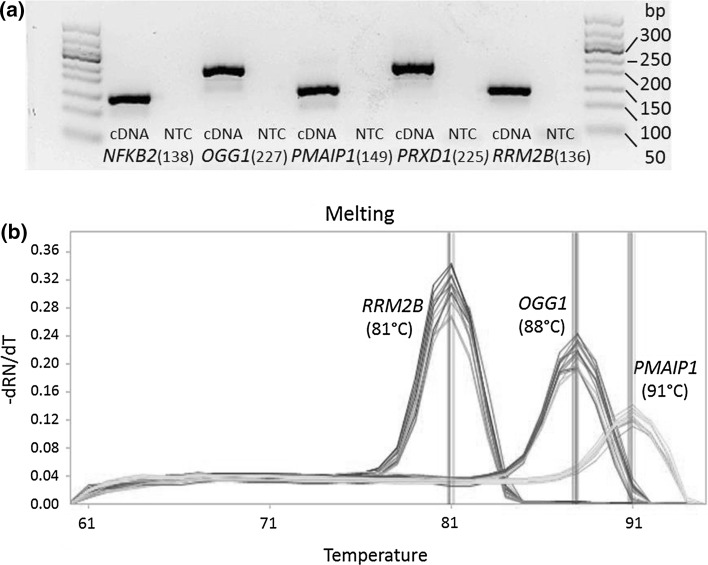
Evaluation of primer specificity. **a** Example of agarose gel electrophoresis analyses for the genes *NFKB2*, *OGG1*, *PMAIP1*, *PRDX1* and *RRM2B* (and included NTCs) with corresponding size of the specific target amplicons. **b** Example of melting curve analyses (BioMark™ HD System) for the genes *OGG1*, *PMAIP1* and *RRM2B* with corresponding melting temperatures of the specific target amplicons

Each primer pair was analyzed by conventional qPCR and displayed highly specific reactions with the target gene. This was demonstrated by a defined melting curve and the verification of the respective target amplicons via size analysis by gel electrophoresis and fluorescence detection. As one example, the electrophoretic analyses displaying the target amplicons of *NFKB2*, *OGG1*, *PMAIP1*, *PRDX1* and *RRMB2* and their corresponding sizes are shown in Fig. 1a. Negative controls, performed with PCR-certified water instead of cDNA, of a few primer pairs showed weak signals in qPCR, but those signals had high *C*_q_ values (>35) and at least a difference of 5 *C*_q_ values to the corresponding positive signal in the cDNA control. Since >5 *C*_q_ values between positive and negative signal would contribute to less than 3 % to the total amount of DNA, this effect can be neglected (Bustin et al. 2009). In a next step, the specificity of each primer pair was verified by high-throughput qPCR on the BioMark™ HD System based on calibration curves. The qPCR temperature program includes a melting curve analysis for each gene under investigation. Additionally, the no-template control (NTC) and a non-reverse-transcribed RNA sample as NoRT control were carried along for the detection of primer-dimer formation or screening for the genomic DNA (gDNA) background, respectively. Melting curve analysis on the BioMark™ HD System displayed the same results as for conventional qPCR. Examples of melting curves applying the BioMark™ HD System of *OGG1*, *PMAIP1* and *RRM2B* are shown in Fig. 1b. The melting temperatures of the gene amplicons were comparable in the two systems. Minor differences of maximal 2 °C could be explained by variation in the settings of the melting curve analyses. The program for the standard qPCR was 65–95 °C with an increase of 1°/5 s, whereas the melting curve applying the BioMark™ HD System was performed from 60 to 95 °C with an increase of 1 °C/3 s. Supplementary material 3 lists sizes and melting temperatures obtained from conventional qPCR and BioMark™ HD System of all target amplicons. This specificity check for the respective primer pairs was performed in every high-throughput qPCR analysis with the BioMark™ HD System, since the melting curve analysis is an integral part of the assay. Additionally, all the controls (NTC, NTC-STA, NoRT) were carried along with each dynamic array run. The signals of the NoRT control were furthermore taken into account in the data analysis (see data analysis and depiction), even though they could in principle be disregarded.

**Fig. 2:**
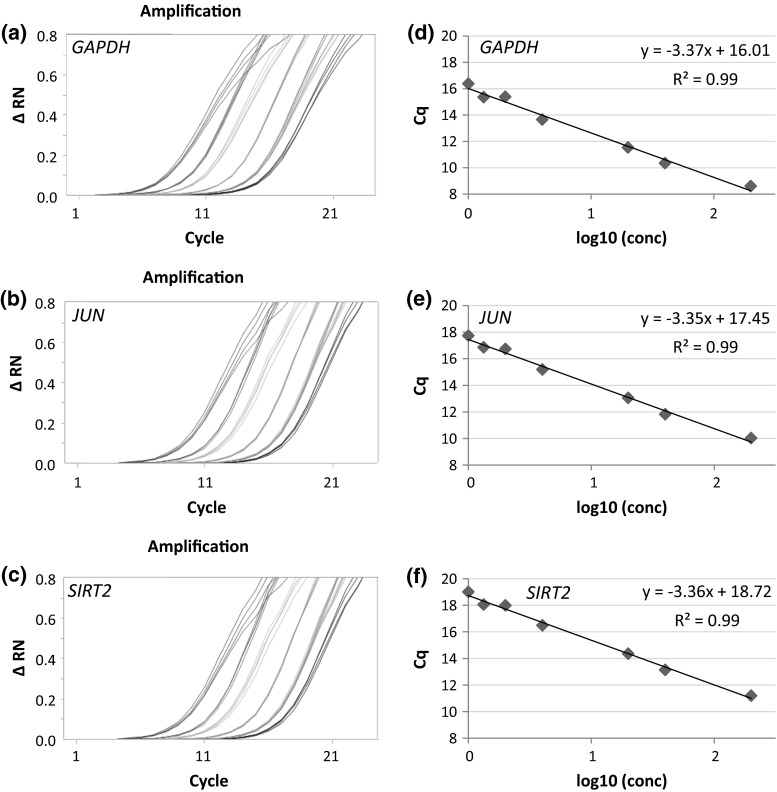
Performance of primer efficiency. Examples of the amplification curves from the calibration performed with Fluidigm dynamic array for **a** *GAPDH*, **b** *JUN* and **c** *SIRT2*. Six serial dilutions (1–200-fold) of standard cDNA are shown. Plot of calibration curves (*x*-*axis* log_10_ of relative template concentration, *y*-*axis* *C*_q_ value of template concentration) with linear regression trend line and correlation coefficient for **d** *GAPDH*, **e** *JUN* and **f** *SIRT2*. Primer efficiency can be calculated from the slope of the calibration curve

PCR amplification efficiency for each primer pair was determined via a calibration curve, established by a serial dilution of commercial human standard cDNA (200-fold serial dilution) and analyzed via the BioMark™ HD System with Fluidigm dynamic array. PCR efficiency was calculated from the slope of the calibration curve (Eq. 1) resulting from linear regression by plotting the logarithm of the relative template concentration on the x-axis against *C*_q_ value on the y-axis with GenEx software. Model amplification curves of the serial dilution and trend lines from linear regression with slope, intercept and correlation coefficient for the genes *GAPDH*, *JUN* and *SIRT2* are displayed in Fig. 2.

1 $${\text{PCR}}\;{\text{efficiency}} = 10^{{( - 1/{\text{slope}})}} {-}1$$

The optimum value for the primer efficiency would be 1.00 (100 %) if the template is doubled within every PCR cycle (Bustin et al. 2009). Even though a 200-fold serial dilution is a narrow range for the determination of PCR efficiency, the correlation coefficients *R* ^2^ of the calibration curves displayed acceptable results over 0.985. Also, the PCR efficiencies revealed adequate values between 90 and 103 % (Supplementary material 4). For 73 out of the 95 genes, primer pairs’ efficiency displayed even very good results between 95 and 100 %. Since basal expression levels for *BRCA1*, *BRCA2*, *CYP1A1*, *GADD45A*, *GPX2*, *PMAIP1* and *RAD51* were very low within purchased human standard cDNA, efficiencies for these genes could not be determined appropriately from the calibration curve due to a too narrow range of diluted samples with acceptable *C*_q_ values. Therefore, a second serial dilution was performed with cDNA derived from A549 cells. As a consequence of higher basal expression levels in this cell line, PCR efficiency could be determined in a precise way for *BRCA1*, *BRCA2*, *GPX2*, *PMAIP1* and *RAD51* (Supplementary material 4). The calibration curves of *CYP1A1* and *GADD45A* still displayed comparatively poor correlation coefficients of 0.971 and 0.917, respectively. This limitation is due to the challenge that common reaction conditions were required for all 95 primer pairs, which, however, appears acceptable. As a consequence, the extent of modulation of those genes is not exactly quantifiable, but rather should be considered as a trend of modulation, and may be confirmed via conventional qPCR. Altogether, a common linear dynamic range for all genes could be identified, and *C*_q_ values of all reported analyses with the BioMark™ HD System were located within this range. For all analyses, a cutoff value of *C*_q_ > 22 was applied, since *C*_q_ values above 23–25 are not reproducible within this system, as downscaling of PCRs into the nanoliter range results in higher Poisson variability due to lower initial template concentrations (Svec et al. 2013). Precision of the qPCR assays was determined by the intra-assay variation of the calibration curves performed in quadruplicate per dilution. Standard deviations (SD) were below 2 % up to 1:50 dilutions and increased to a maximum of 4 % for the higher dilutions, as SD increases with lower template amount.

### Impact of cadmium on gene expression profiles

A549 cells were treated with 10 or 50 µM and BEAS-2B cells with 5 or 10 µM CdCl_2_ for 8 or 24 h, as described for the respective experiments. Concentrations were selected based on cytotoxicity. While 24-h treatment with 50 µM CdCl_2_ showed only low cytotoxicity in A549 cells, reducing both cell number and colony-forming ability to about 70 % (Schwerdtle et al. 2010), cytotoxicity was much more pronounced in BEAS-2B cells. Since they do not form colonies, cell numbers were quantified. They decreased to 85 % in case of 5 µM and to 55 % in case of 10 µM CdCl_2_ after 24-h treatment. No cytotoxicity was seen in case of 8-h treatment at both concentrations.

#### Impact on genes related to (oxidative) stress response

**Fig. 3:**
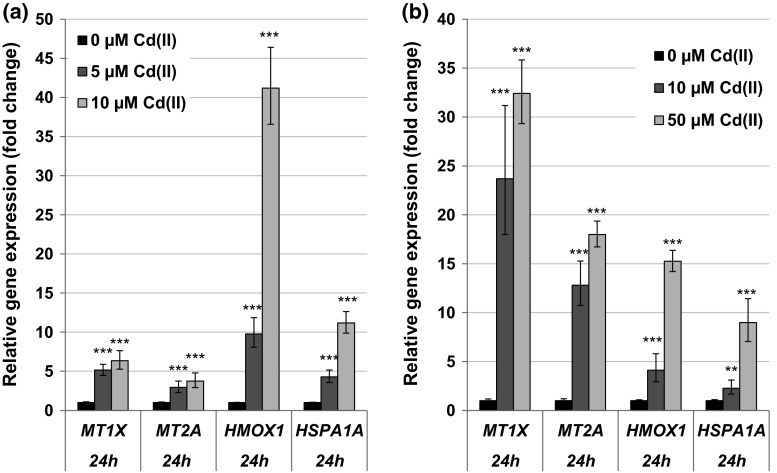
Impact of cadmium on gene expression related to uptake and oxidative stress response. BEAS-2B cells (**a**) or A549 cells (**b**) were treated with CdCl_2_ for 24 h. Shown are mean values of four determinations derived from two independent experiments ± SD. Statistically significant different from control: **p ≤ 0.01, ***p ≤ 0.001 (ANOVA–Dunnett’s *T* test)

**Fig. 4:**
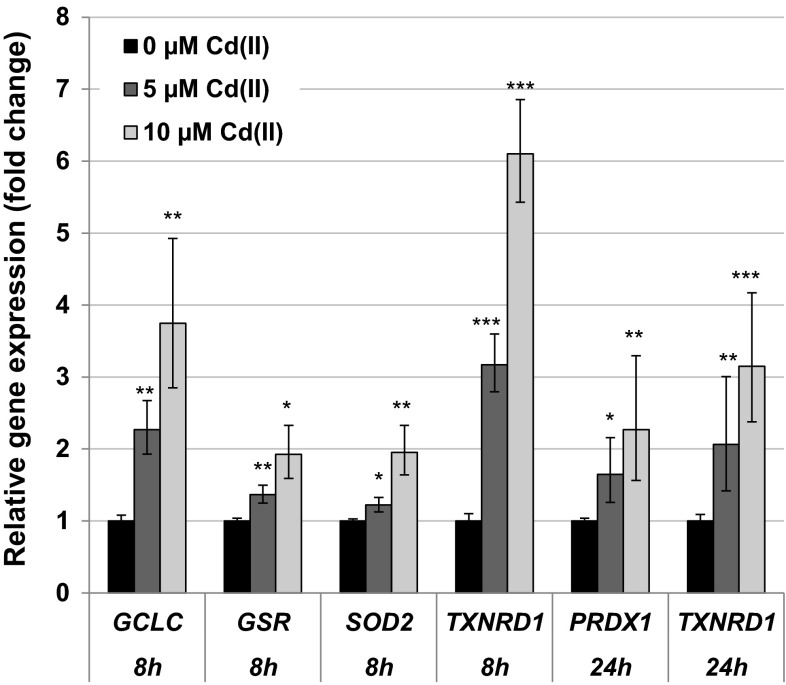
Impact of cadmium on gene expression related to the anti-oxidative defense system. BEAS-2B cells were treated with CdCl_2_ for 8 or 24 h. Shown are mean values of four determinations derived from two independent experiments ± SD. Statistically significant different from control: *p ≤ 0.05, **p ≤ 0.01, ***p ≤ 0.001 (ANOVA–Dunnett’s *T* test)

A pronounced dose-dependent up-regulation of the metallothionein genes *MT1X* and *MT2A* was observed after 8- and 24-h treatment, starting at the lowest concentrations applied. Regarding 24 h, in BEAS-2B cells transcript levels of *MT1X* and *MT2A* were increased up to sixfold and fourfold, respectively (Fig. 3a). In A549 cells, the induction of these genes was even higher, namely up to 30-fold in case of *MT1X* and 18-fold in case of *MT2A* (Fig. 3b). Additionally, an induction of the oxidative stress response system at the transcriptional level was observed in both cell lines after 8- and 24-h treatment; here, stronger effects were observed in BEAS-2B cells. Thus, the transcription of the ROS- and heat shock-sensitive genes *HMOX1* and *HSPA1A* was up-regulated concentration-dependently to a maximum of 40-fold or tenfold, respectively, after 24-h incubation in BEAS-2B cells (Fig. 3a) and 15-fold or ninefold, respectively, in A549 cells (Fig. 3b), again starting at the lowest concentrations. Additionally, genes coding for the glutathione- and thioredoxin-dependent redox system as well as for further anti-oxidant enzymes displayed dose-dependent increased levels of cellular mRNA in BEAS-2B cells (Fig. 4) but not in A549 cells (data not shown). Highest increases were observed in case of *TXNRD1* and *GCLC* after 8 h. Except for *GSR* and *SOD2*, the induction was evident already at the lower concentration of 5 µM CdCl_2_. After 24 h, a duplication of transcript levels was only observed in case of *PRDX1* and *TXNRD1* (Fig. 4); however, at this time point, additionally the mRNA levels of the genes *FTH1, G6PD, GSR, TFRC* and *TXN* were elevated close to twofold at 10 µM CdCl_2_ (data not shown).

#### Impact on genes related to cell cycle regulation and proliferation

**Fig. 5:**
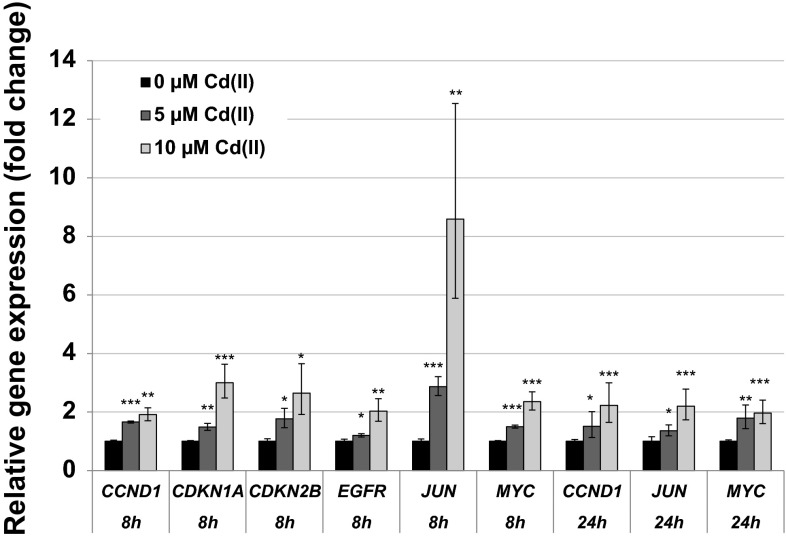
Impact of cadmium on gene expression related to cell cycle regulation and proliferation. BEAS-2B cells were treated with CdCl_2_ for 8 or 24 h. Shown are mean values of four determinations derived from two independent experiments ± SD. Statistically significant different from control: *p ≤ 0.05, **p ≤ 0.01, ***p ≤ 0.001 (ANOVA–Dunnett’s *T* test)

Cadmium induced both growth-promoting and cell-cycle-regulating genes, yielding, however, different patterns at different time points. With respect to mitotic signaling, transcription levels of *JUN* were elevated most pronounced up to eightfold after 8 h. Additionally, cadmium elevated the transcript levels of the proto-oncogenes *MYC* and *EGFR* as well as of the gene *CCND1* coding for the cell cycle progressor cyclin D. However, these inductions were coincident with enhanced mRNA levels of the cell cycle inhibitor genes *CDKN1A* and *CDKN2B* in BEAS-2B cells. After 24 h only the up-regulation of *CCND1*, *JUN* and *MYC* persisted, but was attenuated concerning the strength of induction as compared to 8 h (Fig. 5). Therefore, a pronounced induction was mainly restricted to the higher dose of 10 µM and not evident at 5 µM. In A549 cells, cadmium displayed only small effects on genes involved in cell cycle regulation and mitotic signaling, restricted to the highest concentration of 50 µM; here, after 8-h incubation, transcript levels of *JUN* and, after 24 h, transcript levels of *CDKN1A* coding for the cell cycle inhibitor p21 were up-regulated up to twofold (data not shown).

#### Impact on genes related to apoptosis

**Fig. 6:**
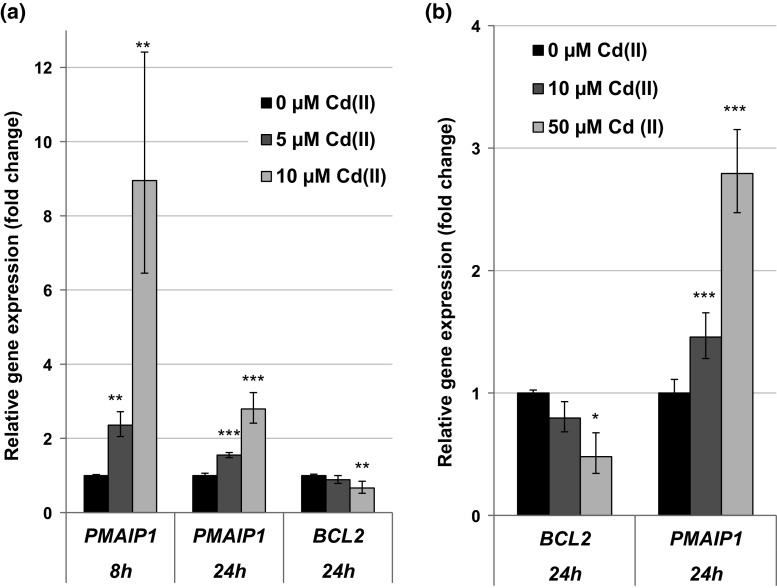
Impact of cadmium on gene expression related to apoptosis. BEAS-2B cells (**a**) or A549 cells (**b**) were treated with CdCl_2_ for 8 or 24 h. Shown are mean values of four determinations derived from two independent experiments ± SD. Statistically significant different from control: *p ≤ 0.05, **p ≤ 0.01, ***p ≤ 0.001 (ANOVA–Dunnett’s *T* test)

Cadmium displayed pro-apoptotic signaling by modulating important genes coding for factors of the intrinsic cascade. Both, in BEAS-2B and A549 cells, the gene *PMAIP1* coding for the pro-apoptotic protein NOXA was transcriptionally induced. However, the strength of the effects differed in the two cell lines. While in BEAS-2B cells *PMAIP1* gene was induced up to ninefold after 8 h and still up to threefold after 24 h, in A549 cells the maximum transcriptional induction was about to threefold. In contrast, the mRNA levels of the anti-apoptotic *BCL2* gene were repressed in both cell lines. This effect was evident in A549 cells at both time points, but in BEAS-2B cells restricted to 24 h. All effects were mainly restricted to the highest concentrations of cadmium applied in the respective cell line (Fig. 6).

#### Impact on genes related to DNA damage response and repair

**Fig. 7:**
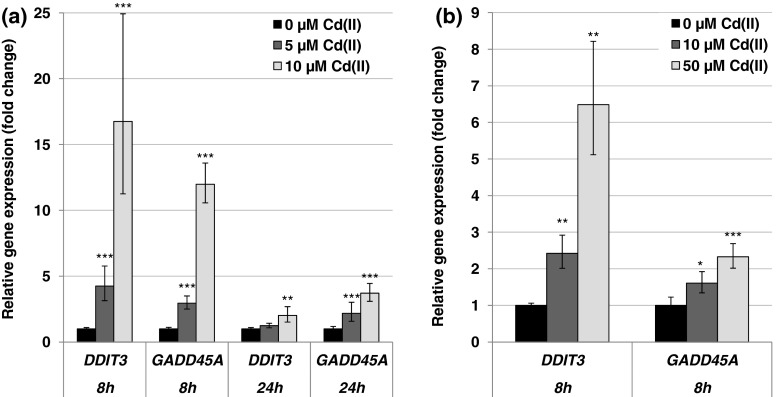
Impact of cadmium on gene expression related to the DNA damage response. BEAS-2B cells (**a**) or A549 cells (**b**) were treated with CdCl_2_ for 8 or 24 h. Shown are mean values of four determinations derived from two independent experiments ± SD. Statistically significant different from control: *p ≤ 0.05, **p ≤ 0.01, ***p ≤ 0.001 (ANOVA–Dunnett’s *T* test)

**Fig. 8:**
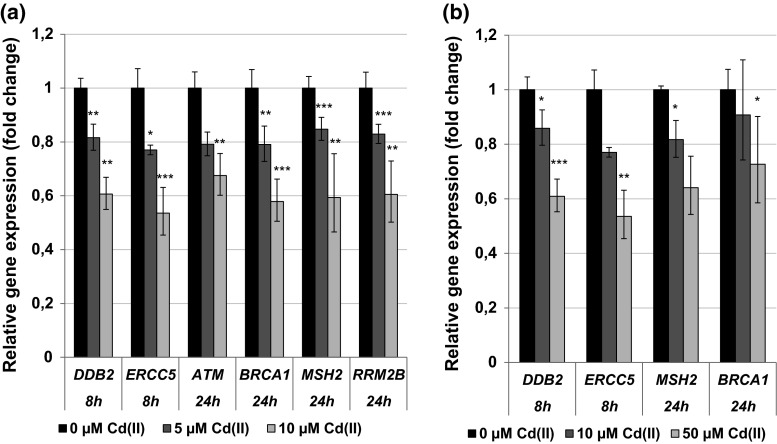
Impact of cadmium on gene expression related to the DNA repair system. BEAS-2B cells (**a**) or A549 cells (**b**) were treated with CdCl_2_ for 8 or 24 h. Shown are mean values of four determinations derived from two independent experiments ± SD. Statistically significant different from control: *p ≤ 0.05, **p ≤ 0.01, ***p ≤ 0.001 (ANOVA–Dunnett’s *T* test)

Concerning the genes coding for the DNA damage response and DNA repair system, transcript levels of the damage inducible genes *DDIT3* and *GADD45A* were highly elevated, predominantly after 8-h treatment of BEAS-2B cells with 10 µM cadmium, reaching levels of up to 17-fold in case of *DDIT3* and up to about 12-fold in case of *GADD45A*. Lower levels of induction were seen after 24 h (Fig. 7a). A similar pattern, but less pronounced, was observed in A549 cells after 8 h (Fig. 7b), with no alterations persisting after 24 h (data not shown). In contrast to the DNA damage inducible genes described above, expression levels of genes coding for proteins involved in DNA damage signaling and DNA repair, such as the signal transducer ATM, the DNA double-strand break repair protein BRCA1, the mismatch repair protein MSH2 as well as several factors important for nucleotide excision repair such as DDB2 and ERCC5, were concentration-dependently repressed in both cell lines; nevertheless, a pronounced reduction in transcript levels occurred only at the higher concentrations (Fig. 8). Altogether, more distinct effects were observed in BEAS-2B cells after 24-h treatment.

## Discussion

The identification of “modes of action” of chemical carcinogens has become a major issue in toxicological risk assessment. While test systems to investigate the induction of DNA damage and mutations have been included routinely within hazard identification of chemical substances, results obtained in molecular and cellular biology have revealed multiple levels of deregulation in tumors which may not be picked up sufficiently in conventional test systems. Thus, maintenance of genomic stability does depend not only on direct interactions with DNA but also on the cellular response to DNA damage, including DNA repair, cell cycle control, apoptosis and stress response signaling. Consequently, with respect to carcinogenic substances, not only direct reactions with DNA bases are of importance, but also the modulation of the stress response and DNA damage response systems. Thus, for example, carcinogenic metal compounds such as cadmium have been shown to interact with DNA repair systems, cell cycle regulation, tumor suppressor functions and cellular signaling [reviewed in Beyersmann and Hartwig (2008), Hartwig (2013)]. This raises the question whether gene expression profiles may be suitable for identifying the multiple interactions involved in chemical carcinogenesis. Within the present study, we present a comprehensive quantitative procedure designed to potentially identify modes of action of chemical carcinogens via high-throughput gene expression analysis, by selection of 95 genes specifically involved in maintaining genomic stability. As one example, we demonstrate that cadmium activated genes coding for the stress response, anti-oxidative defense, mitotic signaling and cell cycle control as well as the intrinsic apoptotic pathway. It further induced damage response genes but down-regulated genes coding for specific DNA repair proteins involved in all major DNA repair pathways. All these interactions mirror the manifold interactions of cadmium supposed to be involved in cadmium-induced carcinogenicity.

As stated in the introduction, there have been manifold approaches during the last years to integrate gene expression profiles in chemical risk assessment. Commonly used techniques for gene expression analyses are frequently based on screening of gene expression changes in the whole genome, such as DNA microarrays or next-generation sequencing. However, results of microarray analyses are semiquantitative and need to be confirmed by PCR. Furthermore, since whole-genome analyses are expensive, they are frequently performed only for a single concentration at one time point. Within the present study, a different approach has been chosen, combining the advantages of quantitative PCR analyses (Derveaux et al. 2010), and the selection of 95 genes specifically related to genomic stability. The analysis of 96 samples in parallel enables dose- and time-dependent investigations of chemicals, an important prerequisite for quantitative risk assessment as opposed to “hazard identification.”

Nevertheless, this procedure also poses a major challenge with respect to its establishment and meaningful application, including the selection of genes of interest, the suitability of all primer pairs, the PCR conditions as well as data evaluation. In a first step, we selected a set of genes involved in maintaining genomic stability for the Fluidigm microfluidic technology to investigate substance-induced changes of expression levels. The reaction of mammalian cells to genotoxic stress can be summarized in a DNA damage response system. Thus, the maintenance of genomic stability is mediated by a complex cellular network comprising the activation of DNA repair systems, cell cycle control and, in case of heavily damaged DNA, apoptosis. Furthermore, DNA lesions may be converted into mutations via the activation of error-prone translesion DNA polymerases. These pathways are tightly regulated also on transcriptional level and modulated as a response to cellular stress (Holbrook and Fornace 1991; Wang 1998; Waters et al. 2009; Zhou and Elledge 2000). The importance of this cellular network for the stability for the genome is further emphasized by the fact that permanent deregulation or inactivation of these signaling pathways is often found in cancer cells (Hanahan and Weinberg 2000, 2011), and chemical-induced modulations may negatively impact genomic stability. Thus, within the present study we selected genes coding for redox-sensitive transcription factors, including respective inhibitors induced as a feedback mechanism upon activation, as well as genes coding for mitotic signaling, cell cycle regulation, DNA repair systems, oxidative stress response, apoptosis and xenobiotic detoxification metabolism.

As compared to conventional RT-qPCR, the major challenge for this high-throughput approach consisted in the establishment of common reaction conditions applicable for all 95 genes under investigation. As one important requisite, much attention was given to the design of every single primer pair. They revealed a high specificity for the respective target genes, assessed via specific melting curves and a correct size of the target amplicons. Additionally, primer efficiencies were convincing in the applied range. Special emphasis was also given to data evaluation. Since manual data analysis of this high-throughput RT-qPCR technique would be difficult to implement and furthermore might be error prone, an appropriate analysis of the raw data was performed with the software Fluidigm real-time PCR analysis. This enabled an efficient evaluation of the quality and reliability of the data. Further data processing was accomplished by using GenEx software. This allowed an additional quality control, the identification of outliers as well as a correction of the *C*_q_ values via subtraction of the gDNA background. This background correction was not absolutely required as the respective NoRT control displayed only negligible signals (Bustin et al. 2009); however, the subtraction added more precision. Since *C*_q_ values above 23–25 are not reproducible on the BioMark™ HD System due to the down-scaling of the qPCR to the nanoliter scale (Svec et al. 2013), data with *C*_q_ values above 22 were excluded from further analysis. A great benefit of the GenEx software consists in the normalization of the data to suitable reference genes. Within this study, the five potential reference genes *ACTB*, *B2M*, *GAPDH*, *GUSB* and *HPRT1* were included; the integrated programs geNorm and Normfinder evaluated their usefulness for each experiment as well as the optimal number and combination of genes which facilitated optimal normalization (Andersen et al. 2004; Vandesompele et al. 2002).

To assess the power and additionally reflect the significance and applicability of the described approach, we performed a time- and concentration-dependent gene expression profiling with cadmium chloride. Two cell lines representing the lung as target tissue of cadmium-induced carcinogenicity were applied: A549 as p53-proficient tumor cell line and BEAS-2B as non-tumor but p53-deficient bronchial epithelial cell line. Overall, the quantitative gene expression analyses reflected known interactions of cadmium related to its genotoxic and carcinogenic potential, namely the induction of metallothioneins, oxidative stress, interactions with DNA and tumor suppressor functions as well as modulations of cellular signaling [reviewed in Hartwig (2010, 2013)], and provided further insight into the cadmium-induced modulation on gene expression related to genomic stability.

The gene expression analyses revealed an intracellular cadmium ion increase by enhanced transcript levels of the metallothionein genes *MT1X* and *MT2A*. Metallothionein genes are up-regulated in response to cadmium ions mediated via the metal-responsive transcription factor MTF-1. The small cysteine-rich metal-binding proteins can effectively bind intracellular free cadmium ions and therefore represent a detoxification mechanism on the one hand, but provoking high intracellular cadmium concentrations on the other hand (Andrews 2000; Hartwig 2010; Karin et al. 1984; Klaassen and Liu 1997; Murata et al. 1999). Induction of the *MT1X* and *MT2A* genes was very distinct in case of all investigated concentrations and time points, but more pronounced in A549 cells when compared to BEAS-2B cells. In contrast, up-regulation of the oxidative stress response system was more pronounced in BEAS-2B cells than in A549 cells. While in A549 cells only *HMOX1* and *HSPA1A* displayed a moderate transcriptional increase, their transcription was far more affected in BEAS-2B cells, and further genes of the anti-oxidative defense system were induced. These differences may be explained by lower levels of intracellular glutathione in BEAS-2B cells as compared to A549 cells (Carmichael et al. 1988; Hatcher et al. 1995; Lian and Wang 2008; Pietarinen-Runtti et al. 1998; Rahman et al. 1996), as well as by a persistent deregulation of the expression of anti-oxidative genes in A549 cells. Thus, the transcription factor Nrf2, which mainly regulates genes coding for anti-oxidative enzymes, is constitutively activated in A549 cells due to a dysfunction of its negative regulator Keap1 (Singh et al. 2006). The induction of oxidative stress by cadmium has been frequently described (e.g., Valko et al. 2006). Even though cadmium ions themselves are not redox active, several indirect effects may account for these observations, namely the release of Fenton-reactive metal ions from metallothioneins (O’Brien and Salacinski 1998), the disturbance of the mitochondrial respiratory chain (Wang et al. 2004) and the inhibition of anti-oxidant enzymes (Valko et al. 2006). The modulation of apoptotic genes was more or less restricted to higher concentrations of cadmium in the respective cell line. Here, a distinct activation of genes coding for the intrinsic signaling cascade indicative for mitochondrial damage was observed at the transcriptional level, characterized by the induction of the pro-apoptotic gene *PMAIP1* coding for NOXA, and a down-regulation of *BCL2*. This pattern agrees with observations described previously for cadmium, disturbing the balance of anti-apoptotic and pro-apoptotic proteins of the Bcl-2 family [reviewed in Thevenod and Lee (2013)]. With respect to cell growth and cell cycle regulation, after 8-h treatment cadmium provoked an up-regulation of the proto-oncogenes genes *MYC*, *JUN* and *EGFR* as well as the *CCND1* gene, coincident with enhanced mRNA levels of the cell cycle inhibitor genes *CDKN1A* and *CDKN2B*. Interestingly, after 24 h only the up-regulation of *MYC*, *JUN* and *CCND1* was still evident in BEAS-2B cells, suggesting a persistent growth stimulus and mitotic signaling. Specifically, c-Jun is part of the transcription factor AP1, an important regulator of many genes involved in cell growth and proliferation, including the up-regulation of *JUN* (Angel et al. 1988; Angel and Karin 1991; Shaulian and Karin 2001). Regarding cadmium-induced carcinogenicity, a persistent up-regulation of proto-oncogenes coincident with a transient enhancement of cell cycle inhibitors indicates a deregulation of cell growth, which has been shown to play a critical role in cancer development (Hanahan and Weinberg 2000). The enhanced induction of the proto-oncogenes *JUN* and *MYC* confirmed and strengthen previous observations, where *JUN*, *MYC* and *FOS* were found to be overexpressed in cadmium-transformed cells [reviewed in Beyersmann and Hechtenberg (1997), Waisberg et al. (2003)]. It is noteworthy that the up-regulation of *JUN* was far more pronounced and only persistent in non-cancer-derived BEAS-2B cells as compared to cancer-derived A549 cells, indicating on the one hand the importance of the use of non-cancer cells for gene expression analysis and on the other hand the potential of our procedure to pick up cell-type-specific reactions. Concerning the DNA damage response system, after 8 h DNA damage inducible genes *DDIT3* and *GADD45A* were distinctly up-regulated, while genes coding for specific proteins in all major DNA repair pathways were down-regulated. These observations correspond to the inhibition of all major DNA repair pathways by cadmium, including nucleotide excision repair, base excision repair, DNA mismatch repair and DNA double-strand break repair [reviewed in Hartwig (2010, 2013)]. Besides the down-regulation of respective genes observed within the present study, interactions by cadmium with distinct DNA repair proteins have been described, e.g., XPA in case of NER, hOGG1 and PARP1 in case of BER as well as the disturbance of important transcription factors, e.g., p53 and Ref-1/APE1, regulating many genes involved in DNA repair [summarized in Hartwig (2013)]. The mechanistic background of the repressed transcript levels requires further investigations, especially with respect to the identification of the involved transcription factors which may be inactivated. One proposed mechanism consists in an interference with redox regulation, for example via interaction with thiol groups in zinc-binding structures of transcription factors. One interesting example is p53, which is usually stabilized upon DNA damage, regulating cell cycle arrest and apoptosis. It serves as a transcription factor, also for some DNA repair genes such as *XPC*. Within the present study, no activation of p53 seemed to take place in either cell line, as indicated from a missing up-regulation of its inhibitor MDM2 (Harris and Levine 2005). With respect to BEAS-2B cells, an activation would not be expected, since p53 is inactivated as a result of the virus transfection applying the SV40 large tumor antigen during immortalization (Levine 2009). Accordingly, expression levels of *MDM2* gene were not altered after cadmium treatment in this cell line (data not shown). However, other known p53-regulated genes, e.g., *GADD45A*, *PMAIP1* or *XPC*, were up-regulated in BEAS-2B cells. These effects are likely due to the activation of other transcription factors such as AP-1, BRCA1, c-Jun, c-Myc or STAT, which are also known to be involved in the regulation of the above-mentioned genes (Gartel and Tyner 1999; Johnson et al. 2013; Perez-Galan et al. 2006; Sheikh et al. 1999; Zhan 2005). Concerning A549 cells, which are in principle p53 proficient, this observation may also reflect the cadmium-induced unfolding of p53 protein via interaction with its zinc-binding domain (Meplan et al. 1999; Schwerdtle et al. 2010), and subsequent loss of its function of as transcription factor. Nevertheless, it cannot be excluded that the down-regulation of DNA repair factors observed in the present study could be due to other mechanisms, such as a decrease in mRNA stability. Furthermore, it has to be considered that changes in gene expression as shown in the present study are only one underlying mechanism potentially contributing to cadmium-induced genomic instability. Other interferences have been observed at posttranslational level, for example via direct interactions with zinc-binding proteins involved in DNA repair, cell cycle control and tumor suppressor functions (Hartwig 2010), the modulation of secondary messengers, e.g., ROS or intracellular Ca^2+^ or of protein kinases enhancing phosphorylation of transcription factors (Waisberg et al. 2003).

Taken together, the investigations reveal novel insights in cadmium-induced modulation of gene expression related to genomic stability, thereby reflecting molecular interactions involved in cadmium-induced carcinogenicity. With regard to time-dependent interactions, effects were most pronounced after 8-h treatment, indicating an acute and strong reaction toward elevated intracellular cadmium ion levels, elevated generation of ROS and DNA damage, presumably due to the inhibition of oxidative defense enzymes. The most obvious difference after 24-h treatment when compared to 8-h treatment concerns the modulation of the cell cycle regulation and proliferation genes: Here, at the early time point, both cell cycle arrest and proliferation-associated genes were affected, while later on only the proliferation stimulus was still visible. Concerning the dose-dependency, several groups of genes, such as MT genes, *HSPA1A* and *HMOX1*, were markedly regulated at both concentrations, while a pronounced induction of genes related to cell cycle regulation and apoptosis, but also the down-regulation of DNA repair genes were mainly restricted to the higher concentrations, displaying beginning to moderate cytotoxic effects in the respective cell lines. Most changes in gene expression were observed both in the cancer cell line A549 and in the non-cancer cell line BEAS-2B, but effects were more pronounced in the latter cells. Altogether, both cell lines display advantages and disadvantages; A549 cells are p53 proficient but as a tumor cell line they have lost some characteristics of the original epithelial type 2 cells and they display some persistent basal signaling deregulation. In contrast, BEAS-2B cells are non-tumorigenic, but p53 deficient due to the immortalization. Therefore, the comparison of two cell lines and the knowledge about their limitations may provide valuable hints for evaluating and interpreting the effects on gene expression.

## Conclusion and outlook

Within the present study, we described the establishment of a test system based on a high-throughput RT-qPCR analysis on BioMark™ HD System that enables the quantitative assessment of gene expression profiles related to genomic stability. In general, it may be applied to analyze basal gene expression levels or their modulations by chemical and physical agents, as shown here for cadmium as a model substance. The designed test system comprises 95 genes and covers important signaling pathways, e.g., DNA damage response, proliferation, apoptosis and oxidative stress response. Evaluation of the primer pairs displayed high specificity for the target genes and appropriate efficiencies in the applied PCR range. The gene expression profiles derived for cadmium-treated cells provided detailed results concerning genes involved in the regulation of the cellular response to uptake, oxidative stress, DNA damage response and apoptosis. It reflected many observations described previously with respect to gene expression (Beyersmann and Hechtenberg 1997; Waisberg et al. 2003), but also with respect to the proposed mechanisms of cadmium-induced carcinogenicity on the functional level, such as inhibition of DNA repair and tumor suppressor reactions, which may be explained by an interferences with cellular redox regulation (Hartwig 2013). By comparing the effects on gene expression between cancer and non-cancer cell lines, distinct differences related to their transformation status were identified. As compared to other approaches investigating the impact of toxic substances on gene expression profiles related to genomic stability, the system described in the present study provides quantitative results without the need of further verification. Due to the high sample number investigated in parallel, it allows dose- and time-dependent investigations as a prerequisite for risk assessment as opposed to hazard identification, as well as the identification of the most sensitive cellular targets within the selected signaling pathways. Thus, in the future, this system may help to predict the mode of action of poorly investigated substances and may serve as the basis for further mechanistic studies on the protein level. Finally, it may be applied to study cell-type- and cell-cycle-dependent interactions as well as the role of specific regulators such as p53. Since the system is flexible with respect to the selection of primers and genes, it is applicable also for other toxicological questions, for example xenobiotic metabolism. Altogether, it may provide a comprehensive basis for in-depth studies on molecular interactions of substances of interest; this needs to be confirmed for further substances with different modes of action.

## Electronic supplementary material

Below is the link to the electronic supplementary material.

Supplementary material 1 (XLSX 13 kb)

Supplementary material 2 (XLSX 25 kb)

Supplementary material 3 (XLSX 13 kb)

Supplementary material 4 (XLSX 19 kb)

Supplementary material 5 (DOCX 161 kb)
