# Supplementary material for: Use of high-throughput RT-qPCR to assess modulations of gene expression profiles related to genomic stability and interactions by cadmium
Source: Arch Toxicol. 2015 Nov 2;90(11):2745–61. doi: 10.1007/s00204-015-1621-7 (PMC5065590; doi:10.1007/s00204-015-1621-7)

**Supplementary figure 5:** RNA integrity. **(a)** 28S rRNA and 18S rRNA bands of 1 µg total RNA on denaturing agarose gel electrophoresis. Intensity of 28S rRNA displays approximately twice the intensity of 18S rRNA, indicating intact RNA. **(b)** Electropherogram from microfluidics-based electrophoresis analysis (2100 Bioanalyzer, Agilent Technologies) with an optimal RIN = 10 and a virtual gel image of 1 µg total RNA.

**(a)**


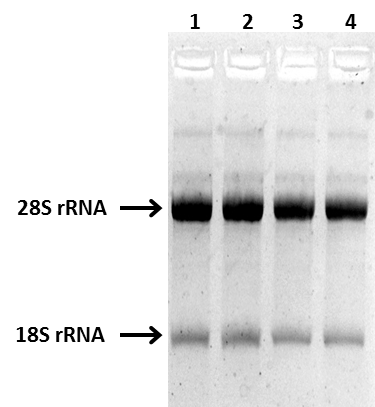


**(b)**


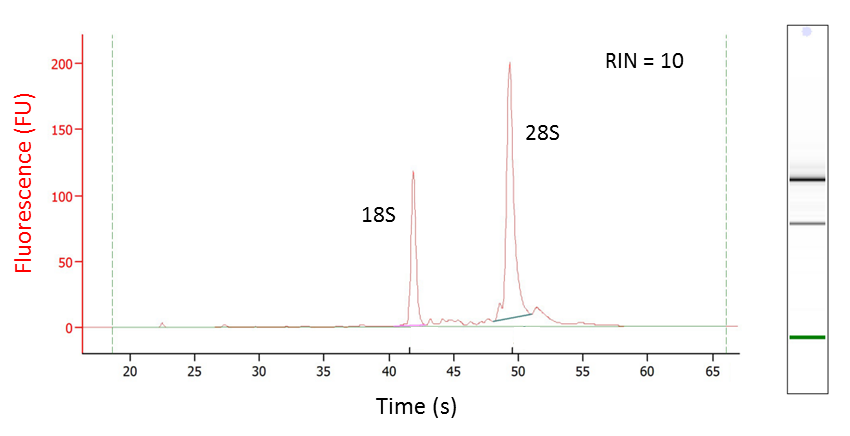

Supplement: Supplementary file 5 — Supplementary material 5 (DOCX 161 kb) [file 204_2015_1621_MOESM5_ESM.docx]
